# Measuring Men’s Gender Norm Beliefs Related to Contraception: Development of the Masculine Norms and Family Planning Acceptance Scale

**DOI:** 10.1007/s10508-021-01941-w

**Published:** 2021-04-05

**Authors:** Sara J. Newmann, Jennifer Monroe Zakaras, Shari L. Dworkin, Mellissa Withers, Louisa Ndunyu, Serah Gitome, Phillip Gorrindo, Elizabeth A. Bukusi, Corinne H. Rocca

**Affiliations:** 1 Bixby Center for Global Reproductive Health, Department of Obstetrics, Gynecology, and Reproductive Sciences, University of California, San Francisco, CA USA; 2 School of Nursing and Health Studies, University of Washington Bothell, Bothell, WA USA; 3 University of Southern California Institute On Inequalities in Global Health, Los Angeles, CA USA; 4 Kenya Medical Research Institute, Nairobi, Kenya; 5 The Department of Public Health, School of Public Health and Community Development, Maseno University, Maseno, Kenya; 6 Department of Obstetrics, Gynecology, and Reproductive Sciences, University of California, Oakland, CA USA; 7 Department of Obstetrics, Gynecology, and Reproductive Sciences, Zuckerberg San Francisco General Hospital, 1001 Potrero Avenue, Unit 6D-14, San Francisco, CA 94110 USA

**Keywords:** Family planning, Men, Gender norms, Kenya, Contraceptive use

## Abstract

Male partner resistance is identified as a key factor that influences women’s contraceptive use. Examination of the masculine norms that shape men’s resistance to contraception—and how to intervene on these norms—is needed. To assess a gender-transformative intervention in Kenya, we developed and evaluated a masculinity-informed instrument to measure men’s contraceptive acceptance—the Masculine Norms and Family Planning Acceptance (MNFPA) scale. We developed draft scale items based on qualitative research and administered them to partnered Kenyan men (*n* = 150). Item response theory-based methods were used to reduce and psychometrically evaluate final scale items. The MNFPA scale had a Cronbach’s α of 0.68 and loaded onto a single factor. MNFPA scores were associated with self-efficacy and intention to accept a female partner’s use of contraception; scores were not associated with current contraceptive use. The MNFPA scale is the first rigorously developed and psychometrically evaluated tool to assess men’s contraceptive acceptance as a function of male gender norms. Future work is needed to test the MNFPA measure in larger samples and across different contexts. The scale can be used to evaluate interventions that seek to shift gender norms to increase men’s positive engagement in pregnancy spacing and prevention.

## Introduction

Male partners who resist contraceptive use undermine women’s reproductive autonomy and contribute to high undesired pregnancy rates in sub-Saharan Africa (SSA). In this region, an estimated 21% of reproductive-age women who wish to avoid pregnancy are not using a modern contraceptive method—the highest proportion in the world (Guttmachernstitute, 2017)—and opposition from male partners is often identified as a factor driving women’s contraceptive nonuse (Apanga & Adam, 2015; Eliason et al., 2013; Imbuki et al., 2010; Palamuleni, 2013). Scholars have thus increasingly called for the inclusion of men in efforts to improve the use of contraception and strengthen reproductive autonomy, particularly given that spousal discussion and joint decision-making have been associated with women’s ability to initiate and continue using contraception when desired (Hartmann et al., 2012; Nketiah-Amponsah et al., 2012; Ntshebe, 2011; Ogunjuyigbe et al., 2009; Tumlinson et al., 2013).

Men may oppose contraceptive use due to a lack of knowledge and fears about side effects or complications (Kabagenyi et al., 2014; Koffi et al., 2018; Schuler et al., 2011). Studies have shown that men are also very concerned about contraception’s perceived potential to bolster women’s reproductive autonomy (Kabagenyi et al., 2014; Mosha et al., 2013) and to undermine their male-dominated decision making in relationships and households (Geleta, 2018; Withers et al., 2015b). This perceived conflict between contraceptive use and men’s ability to conform to their ideas of what an ideal man is consistent with research that has long emphasized the significance of gender norms in shaping health (Fleming & Agnew-Brune, 2015). For example, a growing literature on masculinity in SSA (Dworkin et al., 2012, 2013a; Odimegwu et al., 2013; Shefer et al., 2008; Sideris, 2004; Silberschmidt, 2011; Stern & Buikema, 2013; Wyrod, 2008) and elsewhere (Fleming et al., 2013; James-Hawkins et al., 2019; Jordal et al., 2015; Maternowska et al., 2014; Wentzell & Inhorn, 2014) has illuminated how men’s adherence to masculine ideologies and their navigation of social expectations of manhood can influence their relationship dynamics and behaviors, with concomitant effects on health outcomes for both themselves and their female partners.

Masculine norms have been found to be associated with behaviors that increase sexual and HIV risk, including lack of condom use and multiple sexual partnerships (Fleming et al., 2016); intimate partner violence perpetration (Fleming et al., 2019; Willie et al., 2018); lower rates of HIV testing (Sileo et al., 2018) and engagement in the HIV care continuum (Sileo et al., 2019); low levels of reproductive healthcare utilization (Maternowska et al., 2014); and contraceptive nonuse (Maternowska et al., 2014; Withers et al., 2015a, 2015b). Amplifying these effects are societal changes, such as gains in women’s rights and decreases in employment and economic stability, that can bolster anxiety about an inability to achieve prevailing expectations of manhood and further drive resistance to behaviors perceived to be out of sync with male gender norms (Dworkin et al., 2012; Morrell, 2002; Silberschmidt, 2011; Shefer et al., 2008; Sideris, 2004; Wyrod, 2008).

Involving men and actively addressing their views about masculinity and gender equality could thus have the potential to decrease their resistance to gains in women’s reproductive autonomy and encourage their positive involvement as partners and allies (Dworkin, 2015; Dworkin et al., 2011). To this end, sexual and reproductive health (SRH) programs are now being called on to constructively address the role of gendered power relations and male gender norms in SRH behaviors and outcomes (Barker et al., 2010; Starrs et al., 2018). Gender sensitive and transformative interventions that seek to change gender inequality and male attitudes and behaviors have been found to be effective in the HIV and gender-based violence fields (Dworkin, 2015; Dworkin et al., 2013b); however, relatively few interventions have attempted to directly shift masculine norms toward more acceptance of contraceptive use and working with a female partner to achieve reproductive preferences (Ghanotakis et al., 2017; Schulermm et al., 2015; Shattuck et al., 2011; Wegs et al., 2016), and a recent systematic review has highlighted how few rigorous intervention studies have been conducted in this area (Ruane-McAteer et al., 2019). Moreover, as SRH efforts increasingly intervene on the relationship between deep-seated beliefs about masculinity and male contraceptive resistance, it is imperative to be able to evaluate the effects of these interventions. In a recent review of women’s empowerment and gender-related measures used in family planning and maternal health program evaluations, Mandal et al. (2017) described the dearth of validated measures and the need for rigorous measures of gender norm change.

The few SRH interventions that have attempted to address masculine norms related to family planning assessed outcomes using measures of gender equitable attitudes (using or adapting the Gender Equitable Men [GEM] scale) (Ghanotakis et al., 2017; Schuler et al., 2015; Shattuck et al., 2011) and women’s empowerment (via CARE’s WE-MEASR tool) (Wegs et al., 2016). In our work, we have found that men’s contraceptive resistance is not fueled exclusively by their endorsement of gender inequality but also by their fears of a loss of masculine identity (Withers et al., 2015a, 2015b). Yet, we are not aware of a psychometrically tested instrument that has been systematically and rigorously designed to measure changes in the ways men see women’s contraceptive use as undermining or bolstering their sense of themselves as men. Thus, a masculinities-informed understanding of men’s reactions to contraception is urgently needed.

We implemented a pilot study in Kisumu County, western Kenya, to evaluate a gender-transformative intervention to shift masculine norms related to male contraceptive resistance toward greater acceptance of contraception. To investigate the intervention’s effect, we used formative research and baseline data to develop and evaluate a novel, masculinity-driven quantitative instrument to measure men’s acceptance of contraception—the Masculine Norms and Family Planning Acceptance (MNFPA) scale. This paper describes the development and preliminary evaluation of the MNFPA scale, the associations between MNFPA scores and the perceived ability and willingness to accept a female partner’s contraceptive use, and the relationship between MNFPA scores and current contraceptive use.

## Method

The creation of the MNFPA scale was informed by the construct modeling approach to scale construction (Wilson, 2005). This method entails an iterative process that begins with delineating a conceptual model, situating the construct within that model, clearly defining the construct, designing items to assess the construct based on qualitative data, and finally using a statistical measurement model to relate item responses back to the construct.

Our team carried out extensive formative research over a six-year period among men, women, couples, and healthcare providers in southwestern Kenya that enabled us to flesh out the factors that shape men’s contraceptive resistance and acceptance. In concert with a large-scale cluster randomized trial evaluating the impact of integrating contraceptive care into HIV services (Grossman et al. 2013), this research aimed to understand perceptions, attitudes, decision-making, and relationship dynamics related to planning a family and contraceptive use (Harrington et al., 2012, 2013; Newmann, 2010; Newmann et al., 2013a, 2013b; Patel et al., 2014; Steinfeld et al., 2013; Tao et al., 2015; Withers et al., 2013, 2015a, 2015b). In particular, we investigated gender power dynamics within couples related to contraceptive use and, relatedly, men’s perspectives on contraceptive use and male involvement in women’s health issues—areas underrepresented in the literature at the time.

Our qualitative research confirmed that many women were structurally and interpersonally disempowered relative to men, feared male resistance to contraception, and faced difficulty discussing pregnancy spacing and contraceptive use with their male partners. These factors contributed to covert and nonuse of contraception (Harrington et al., 2012, 2013; Newmann, 2010; Newmann et al., 2013a, 2013b; Tao et al., 2015; Withers et al., 2013). Our findings suggested that gender norms played a key role in these relationship dynamics around contraceptive use. While many men perceived advantages to using contraception, particularly the financial benefits of smaller families and improved health and well-being of female partners and families with birth spacing (Withers et al., 2015b), resistance was pronounced. A key theme that emerged was fear that contraceptive use and limiting family size undermined their male roles in families and communities (Newmann, 2010; Withers et al., 2015a, 2015b). Specifically, contraception was perceived to conflict with masculine norms concerning fertility (male duty to father children); land (paternal family lineage and land inheritance); wealth (perceived wealth and social status of larger families); and sexuality (male discomfort with discussing sex), as well as with patriarchal norms related to male decision-making power in sexual relationships. These concerns were amplified by frustrations over the inability to fulfill traditional expectations of masculinity regarding male economic providership and household authority; many men described grappling with rapidly changing gender roles due to women’s increasing labor force participation and involvement in family decision-making. Finally, men felt unknowledgeable about family planning, fueling concerns about side effects and complications, due to their exclusion from the “female domain” of contraceptive services (Newmann, 2010; Patel et al., 2014; Withers et al., 2015a, 2015b).

### Conceptual Model and Definitions

In developing our conceptual model representing male acceptance of contraception, we drew from two gender-related theoretical frameworks and two behavior change theories that were consistent with our formative research findings. Connell’s (1987) theory of gender and power (TGP) characterizes the gendered relationships between men and women as being a function of social structures and norms that shape gender roles and gender power dynamics. This theory has been used to highlight the ways in which these gendered relationships can increase women’s vulnerability to adverse health outcomes (Wingood & DiClemente, 2000). The second theory, Courtenay’s (2000) constructionist theory of masculinity, posits that ideas about what it means to be a man are locally and regionally constructed, can change, and can therefore be intervened upon to improve gender equity and health. Courtenay argued that men organize their beliefs and actions with reference to the way they perceive men ought to be, and these beliefs and actions can negatively impact their own health and that of their partners. We operationalized TGP and Courtenay’s theory through a focus on the role that male partners can play in influencing women’s contraceptive behaviors, particularly through their endorsement of social norms regarding contraception and masculinity. Finally, in delineating the hypothesized variables that drive male contraceptive acceptance, we drew from social cognitive theory (SCT) (Bandura, 1989) and the theory of planned behavior (TPB) (Ajzen, 1991) to include contraceptive self-efficacy and intention in order to address the importance of the social environment (SCT) and intention (TPB) in learning and performing new behaviors.

**Fig. 1:**
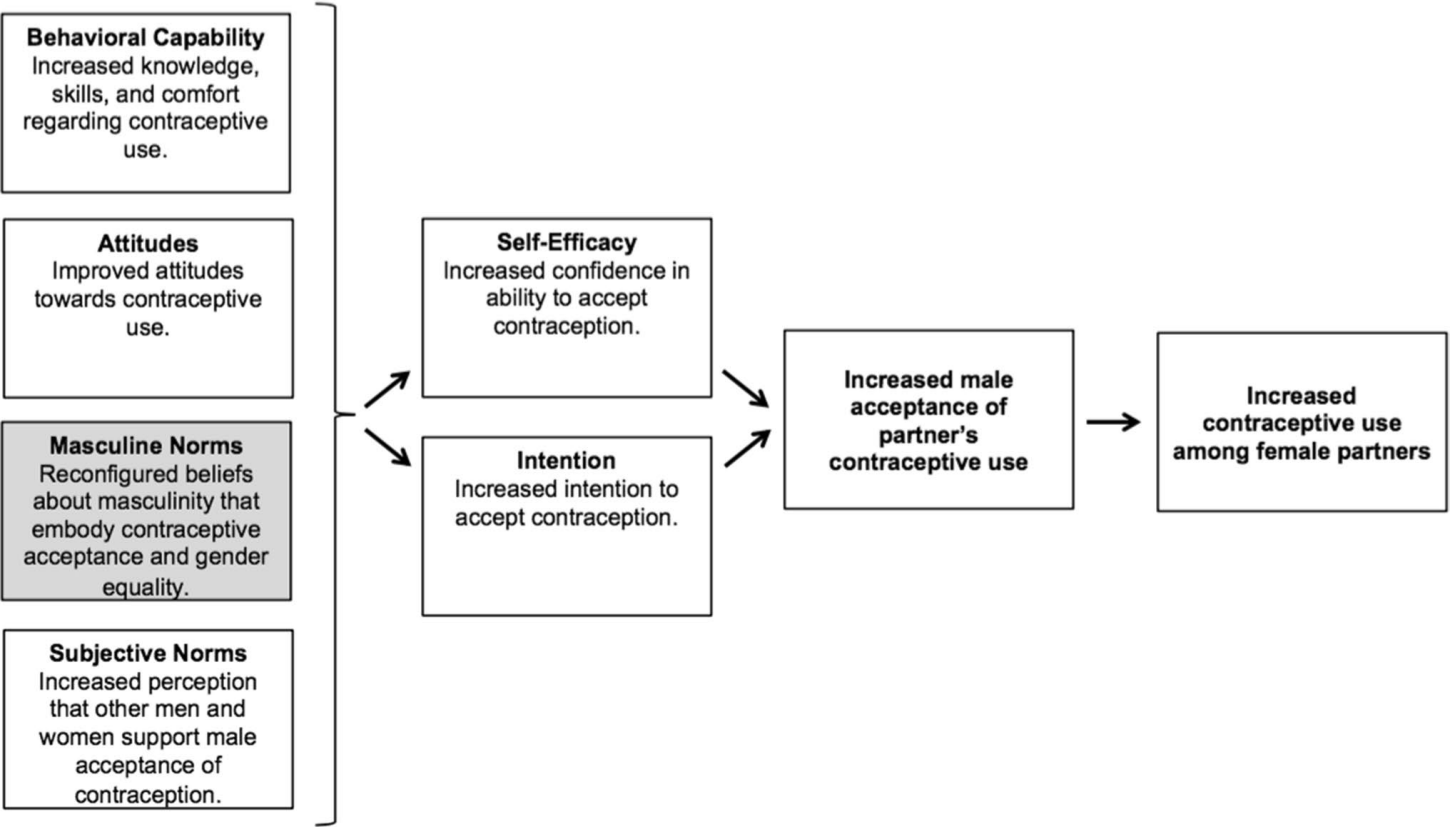
Conceptual framework delineating how masculine norms contribute to male acceptance of contraception

In our resulting conceptual framework (Fig. 1), we centered and defined male acceptance of contraception as (1) respecting a female partner’s contraceptive decisions, (2) approving of her contraceptive use, (3) agreeing to male condom use, and/or (4) being positively involved in contraceptive decisions, including communication and joint contraceptive decision-making with a female partner, accompanying a partner to a clinic that offers contraceptive services, and/or participating in contraceptive counseling. We posited that male contraceptive acceptance was shaped by both self-efficacy and intention to use contraception; these variables, in turn, were influenced by behavioral capability (knowledge and skills related to contraception), attitudes toward contraception, masculine norms regarding contraception, and subjective norms.

Given the prominent role that gender norms played in men’s contraceptive resistance in our formative research and the lack of existing instruments to measure masculine norms related to contraception, we focused our instrument development on the masculine norms construct. Specifically, we defined this construct, MNFPA, as reconfigured beliefs about masculinity that embody contraceptive acceptance and gender equality. Per the qualitative work, we viewed masculine norms as perceptions of male duties and expectations regarding sexuality and reproduction, land and wealth, and decision-making and household authority.

### Item Development

Drawing directly from comments and insights from our qualitative data, we drafted items to capture manifestations of masculine norms in the context of contraceptive use and the planning of one’s family. The study team, including Kenyan co-investigators, iteratively reviewed, discussed, and modified items to ensure that they were clear, relevant to the cultural context, and encompassed what we felt was the full scope of participants’ perspectives represented in the data. To further ensure content validity, we consulted three colleagues with expertise in measurement development and male SRH engagement to review and provide feedback on the items.

Our item formation process resulted in 44 potential items with four Likert response categories that included strongly agree, agree, disagree, and strongly disagree; responses were coded depending on the item’s direction, so that the highest response category reflected the highest contraceptive acceptance. While the scale was designed to measure male acceptance of contraception and the planning of one’s family, we used the term “family planning” in the scale title and items because, in our study region, men and women most commonly use the term “family planning” to refer to “contraception.”

Item comprehension was assessed through one-on-one cognitive interviews with 10 men in Kisumu County, Kenya, before initiating the quantitative field test. Male interview participants were asked to reflect on items as they completed them, indicating to the interviewer how they interpreted items to ensure that items were understood as intended. Participants also gave feedback on item clarity and preferences for response categories. Draft items were revised as needed based on the cognitive interview feedback.

### Quantitative Field Test

The 44 revised draft items were administered as a field test to 150 men in two communities of similar size and demographic profile in Kisumu County. Located in southwestern Kenya on the border of Lake Victoria, Kisumu is a semi-urban county where the primary occupations are in agriculture, unskilled manual labor, or domestic service (Kenya National Bureau of Statistics and ICF International, 2014). Nearly one quarter of married women aged 15–49 years have an unmet need for contraception, and the HIV prevalence, at 20%, is 3.4 times higher than the national prevalence (Kenya National AIDS Control Council, 2016; Kenya National Bureau of Statistics and ICF International, 2014).

We purposively sampled and recruited Dholuo-speaking, partnered men of reproductive age using direct contact from male-focused social venues, such as soccer matches and bicycle taxi termini, or from their homes. Each participant gave informed voluntary written consent before being enrolled. Trained interviewers who were recruited from the local community administered the surveys in the Dholuo language on paper and in person at the participants’ home or preferred location. Study approval was granted by the Committee on Human Research at the University of California, San Francisco and the Scientific and Ethics Review Unit at the Kenya Medical Research Institute.

### Psychometric Evaluation

We used item response theory-based methods (IRT), in concert with classical test theory approaches, to reduce the item set and evaluate the psychometric properties of the final scale, following guidelines for the psychometric testing of new instruments (AERA, APA, & NCME, 2014; Wilson, 2005). IRT is a leading statistical approach to the development of latent variable measures, including participant reported outcomes (de Boeck & Wilson, 2004; Embretson & Reise, 2000; Hays et al., 2000; Wilson et al., 2006). Preliminary item responses were fit to a unidimensional partial credit IRT model for polytomous items using ACER ConQuest software (Adams et al., 2016; Masters, 1982). We assessed model fit using weighted mean square statistics, removing items falling outside of 0.67–1.33 as a guideline (Wright & Masters, 1982), and internal consistency reliability with the separation reliability coefficient. To assess internal structure validity, we investigated whether respondents endorsing higher categories on each item had higher overall scale scores. To ensure the scale met the assumption of unidimensionality for the IRT model, we supplemented IRT analyses with exploratory factor analysis and ensured items all loaded onto one factor with an eigenvalue of ≥ 1.0 (Kaiser, 1960). We also calculated the Cronbach’s (1990) α. At this stage, we removed items that reduced the scale’s reliability, those with highly skewed distributions, and those with conceptual overlap with other, better performing items. Given that many of the items had responses reflecting high levels of contraceptive acceptance, we collapsed the two lowest response categories.

In evaluating the final instrument, we again fit responses to the reduced item set to the partial credit model and assessed model fit, internal consistency reliability, and internal structure validity. To garner evidence for external validity, we evaluated the association between MNFPA scores and two constructs we hypothesized to be affected by masculinity norms about contraceptive use: contraceptive self-efficacy and contraceptive intention (Fig. 1). The self-efficacy and intention items were developed based on the formative research and were comparable to self-efficacy and intention questions used in prior research. We used ordinal logistic regression to test the association between MNFPA scores and responses to questionnaire items probing these two constructs. Given that intention responses would be dependent on pregnancy desire, the intention analysis was restricted to men who reported not wanting pregnancy within the next three months. We also assessed differential item functioning (DIF) to determine whether any scale items performed differentially among men by age (< 30 vs. ≥ 30), education (up to primary vs. more than primary), number of children fathered (< 3 vs. ≥ 3), and HIV status (negative vs. positive). We used item-by-group parameter effect size of ≥ 0.6 logits as suggestion of DIF (Longford et al., 1993; Paek, 2002).

To begin to explore the role of MNFPA in shaping contraceptive use in the longitudinal data of our gender-transformative pilot study, we examined sociodemographic factors associated with MNFPA scores and then the association between MNFPA scores and reported contraceptive use cross-sectionally. We used multivariable linear regression to examine key sociodemographic variables associated with MNFPA scores and multinomial logistic regression to assess the association of MNFPA scores with contraceptive method use (dual hormonal/sterilization and condom, hormonal/sterilization only, condom only, neither). Given our limited sample size and the exploratory nature of analyses, we reported significance to the *p*-value < .10 level.

## Results

Respondents were on average 33 (range: 20–62) years old, and 40% had more than a primary school education. Nine percent were in a polygamous marriage or relationship, and 61% had fathered three or more children. Seventeen percent reported that they were HIV positive or did not know their status (Table 1). Overall, participants demonstrated relatively high levels of contraceptive acceptance in response to MNFPA items. For instance, 75% percent of men strongly disagreed that women who initiate contraceptive discussions are trying to take power away from men. At the same time, only 7% of respondents disagreed or strongly disagreed that a woman should seek permission from her male partner to use a contraceptive method. About one half of the men strongly agreed or agreed that a man has the final say in contraceptive decisions (data not shown).

**Table 1:** Male sample characteristics (*n* = 150)

Characteristic	*n*	%
Age, mean years, SD (range 20–62)	32.7 (8.4)	
Polygamous marriage/relationship	14	9.3
Has more than a primary school education	60	40.0
*Number of children fathered*		
0	6	4.0
1	23	15.3
2	30	20.0
3	32	21.3
4	18	12.0
5 +	41	27.3
*HIV serostatus*		
HIV negative	125	83.3
HIV positive	20	13.3
No test or don’t know	5	3.3
*Desired fertility timing*		
Wants no more children	36	24.0
Wants a child in > 2 years	68	45.3
Wants a child within 2 years	46	30.7
Discussed family planning with main partner, last 6 months	96	64.0
*Most effective contraceptive method currently using*^a, b^		
None	16	11.7
Abstinence, withdrawal, rhythm, lactation	8	5.8
Condom, other barrier	20	14.6
Pills	12	8.8
Injectable	39	28.5
Implant	41	29.9
Female sterilization	1	0.7
*Current dual contraceptive-condom use*^a^		
Dual^c^	33	24.1
Hormonal or sterilization only^b, d^	61	44.5
Condom only	19	13.9
Neither	24	17.5

^a^ Excludes participants desiring pregnancy within 3 months; *n* = 137

^b^ No participant reported use of an intrauterine device (IUD) or male sterilization

^c^ Dual use of male condoms and a hormonal method or sterilization

^d^ Methods include female and male sterilization, intrauterine device (IUD), implant, injectable, and pills

The 10 final scale items fit a unidimensional partial credit item response model and had a separation reliability of 0.67 (Table 2). Most scale items met criteria for internal validity, including correspondence between each item’s response categories and overall scale scores. For three items, those providing the lowest responses (strongly agree or agree together) had slightly higher average MNFPA scores than those answering disagree (Items 2, 3, and 8). Items performed non-differentially by age and number of children. We identified some evidence of DIF by education and HIV status for Item 8: men with more than a primary education and HIV-negative men were more likely to disagree with the statement that “A man has the final say in family planning decisions” compared to those with a primary education or less, or those who were HIV-positive, respectively.

**Table 2:** MNFPA scale reliability and properties

	Mean (SD)	Item response model (Separation reliability: 0.67)		Classical test (Cronbach’s α: 0.68)	
MNFPA Item		Item fit	Item difficulty/location	Item-total correlation	Factor loading
(1) A man who has many children is a strong male	1.45 (0.81)	0.98	− 0.11	0.60	0.43
(2) Men with more than one female partner should avoid using family planning methods	1.65 (0.63)	1.06	− 0.73	0.45	0.32
(3) A man who undergoes vasectomy is a weak male	1.39 (0.78)	1.07	− 0.07	0.51	0.33
(4) A woman who wants to use a family planning method is undermining her male partner as the head of the household	1.59 (0.66)	0.95	− 0.60	0.61	0.69
(5) Women who use family planning methods are taking power away from men	1.60 (0.71)	0.91	− 0.45	0.61	0.66
(6) Women who initiate a discussion about using family planning methods with their male partners are trying to take power away from men	1.68 (0.61)	0.88	− 0.81	0.62	0.65
(7) The decision to use a family planning method should be made solely by the man	1.59 (0.63)	1.01	− 0.70	0.51	0.41
(8) A man has the final say in family planning decisions	0.84 (0.87)	1.30	0.96	0.38	0.15
(9) A woman should seek permission from her male partner to use a family planning method	0.12 (0.45)	1.05	2.65	0.26	0.14
(10) Women who use family planning methods are unfaithful	1.43 (0.75)	1.02	−0.31	0.55	0.46

For all MNFPA items, disagreeing indicates higher levels of male acceptance of contraception. Items were scored: 0 = strongly agree or agree, 1 = disagree, 2 = strongly disagree

Item fit and difficulty/location (in logits) are derived from a partial credit item response model. Item fit is the weighted mean-squared fit *t-*statistic

**Fig. 2:**
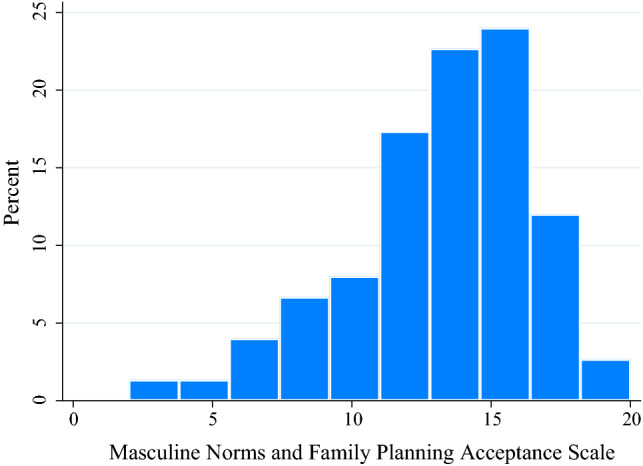
Distribution of MNFPA scores

Using summed raw responses, average scores were 13.3 (SD: 3.5, Min: 2, Max: 20) on the 0–20 scale; the distribution was near normal with a left skew (Cronbach’s α: 0.68) (Fig. 2). All items loaded onto a single factor with eigenvalue > 1.0. There were no significant differences in average MNFPA scores by any sociodemographic characteristics.

As hypothesized, men scoring higher on the MNFPA scale exhibited higher levels of contraceptive self-efficacy and intention (Table 3). For each increased point on the MNFPA scale, there was a significant increase in perceived ability to use contraception even when faced with public ridicule (OR = 1.18, 95% CI: 1.04, 1.33); increasing MNFPA scores were negatively associated with finding it difficult to accept a main partner’s contraceptive use because it challenges the respondent’s role as a man in his household (OR = 0.80, 95% CI: 0.72, 0.88) and in his community (OR = 0.83, 95% CI: 0.75, 0.91). Additionally, MNFPA scores were significantly associated with men’s intention to use contraception to space births if a main partner wished (OR = 1.20, 95% CI: 1.04, 1.39) and negatively associated with expecting to become angry if a main partner wanted to use a contraceptive method (OR = 0.83, 95% CI: 0.74, 0.93).

**Table 3:** Association between MNFPA scores and contraceptive self-efficacy and intention

Item	OR (95% CI)	*p*-value
*Self-efficacy to accept contraception*		
I feel confident that my main partner and I could use a family planning method, even if other people in my community ridiculed me.^a^	1.18 (1.04, 1.33)	.009
I find it difficult to accept my main partner using a family planning method because it challenges my role as a man within my household.^b^	0.80 (0.72, 0.88)	< .001
I find it difficult to accept my main partner using a family planning method because it challenges my role as a man in my community.^b^	0.83 (0.75, 0.91)	< .001
*Intention to accept contraception*		
If my main partner wanted to use a family planning method in order to plan (space) births, I would agree with her.^a, c^	1.20 (1.04, 1.39)	.010
I would be angry if my main partner wanted to use a family planning method.^b, c^	0.83 (0.74, 0.93)	.002

^a^ Response options for the dependent variable included 0 = strongly disagree, 1 = disagree, 2 = agree, and 3 = strongly agree

^b^ Response options for the dependent variable included 0 = strongly agree, 1 = agree, 2 = disagree, and 3 = strongly disagree

^c^ Excludes participants desiring pregnancy within 3 months; *n* = 137

Of men who did not desire pregnancy within three months, a majority (58%) reported their partner was currently using a hormonal contraceptive method or sterilization, most commonly the implant (30%) and injectable (29%) (Table 1). Overall, 24% reported using male condoms and either a hormonal method or sterilization, 45% reported using a hormonal method or sterilization alone, and 14% used male condoms alone, while 18% used neither. MNFPA scores among these groups were 13.2 (dual), 14.0 (hormonal or sterilization alone), 13.9 (condom alone), and 13.0 (neither) (data not shown). There were no significant differences in contraceptive use by MNFPA score (Table 4).

**Table 4:** Multivariable multinominal logistic regression model for dual hormonal contraceptive or sterilization and condom use

	Contraceptive use^a^ (reference: No method) aRR (95% CI)		
	Dual use: Condom and hormonal or sterilization	Hormonal or sterilization	Condom only
MNFPA score	1.0 (0.9, 1.2)	1.1 (1.0, 1.3)	1.1 (0.9, 1.3)
Age, in years	1.0 (0.9, 1.0)	0.9 (0.9, 1.0)	0.9 (0.9, 1.0)
More than primary school education	0.8 (0.3, 2.4)	0.5 (0.2, 1.3)	0.4 (0.1, 1.5)
HIV positive	4.5 * (0.8, 25.8)	0.4 (0.1, 3.4)	5.7 * (0.9, 37.1)
Wants no more children	1.0 (0.3, 3.5)	0.7 (0.2, 2.2)	0.9 (0.2, 4.3)

^*^ p < .10

^a^Excludes participants desiring pregnancy within 3 months; *n* = 137

## Discussion

The MNFPA scale is the first psychometrically assessed tool to measure male contraceptive acceptance within the context of masculine norms. The rigor of our scale development process, including the theory-informed development of a conceptual framework, careful creation of items based on extensive formative research, and use of advanced scale evaluation methodologies, supports the construct validity of the MNFPA measure. Additionally, the association between contraceptive self-efficacy and intention and scale scores shows alignment with our conceptual model and further supports the external validity of the measure. Finally, our efforts to identify the specific male gender norms most strongly tied to contraceptive use provides useful guidance to other researchers and program developers who are seeking to understand and measure these norms in their SRH work with men.

Our finding that MNFPA scores were not significantly associated with reported use of contraception highlights the complexity of the relationship between men’s acceptance of contraception and actual couples’ contraceptive use. Myriad factors, male contraceptive acceptance among them, influence a woman’s use of contraception, including her personal attitudes toward contraception, pregnancy preferences, and access to contraceptive services. It is also likely that one’s social networks influence whether their contraceptive acceptance translates into actual use (Lapinski & Rimal, 2005). For example, perceiving disapproval from other men in the community may deter a man from acting to initiate a method. The role of social networks in supporting or undermining an individual’s reproductive preferences is well documented (Igras et al., 2017). Our small sample and high reported contraceptive use may have also hindered our ability to detect an association between contraceptive acceptance and use. Additionally, our cross-sectional results may be affected by time-dependent confounding; women in strongly gendered relationships may seek the use of contraception at higher rates, or men’s acceptance of contraception may be negatively affected by a partner’s contraceptive use, particularly if the method was initiated without the man’s knowledge, which is a reported source of strain and distrust in relationships (Harrington et al., 2016; Koffi et al., 2018).

HIV-positive men in our sample were more likely to report condom use—with or without a hormonal method—than HIV-negative men. Although our small sample size precluded our formal evaluation of interaction, it is likely that the relationship between MNFPA and contraceptive use also differs by HIV status. Studies have shown that HIV status can affect fertility desires (Wekesa & Coast, 2014; Withers et al., 2013), and in a high HIV prevalence area like western Kenya, the imperative for HIV prevention combined with societal pressure and personal desire to restrict or pursue future childbearing may shape contraceptive attitudes and behaviors in complex ways. Moreover, male contraceptive acceptance may influence the use of different contraceptive methods in ways not necessarily associated with the effectiveness of the method for pregnancy prevention, particularly if men and women prioritize other considerations in their method choice (such as HIV prevention).

In a recent review of the measurement of social norms affecting modern contraceptive use, all but two studies used condom use as their outcome of interest, demonstrating a gap in the evidence base concerning the relationship between norms and hormonal methods of contraception (Costenbader et al., 2017). Our scale items did not specify barrier or hormonal methods, so our results may not have captured nuances in the relationship between acceptance and use for these two method groups. Additionally, our conceptual model and scale items did not differentiate between methods that women control versus those that men themselves can use (withdrawal, condoms, vasectomy), and it is possible that the influence of male contraceptive acceptance, especially against the backdrop of masculinity, may play out differently for female and male methods. Better understanding these potentially different causal pathways can also help broaden SRH interventions with men to address them as contraceptive users in their own right, rather than primarily as a conduit for increasing female use (Hardee et al., 2017). Continued research is thus urgently needed, including longitudinal assessments with different populations of men and women, to tease apart the complex relationship between contraceptive use and male gender norms.

While strengthened by a careful and rigorous scale development process, our study also had a number of limitations. Our scale items were developed for use in western Kenya, and they may not be generalizable to other areas and populations. Due to feasibility constraints, we integrated this measure development work into a small, ongoing longitudinal study; we were unable to reassess item performance in a second, separate sample. Future work utilizing the MNFPA scale should reassess its psychometric performance, including differential item functioning, in different and larger samples.

Scale responses may have been vulnerable to social desirability bias. In an attempt to mitigate this type of bias, we employed local interviewers who were unknown to participants. High MNFPA scores may have also been the result of our purposive sampling procedures, which could have recruited men who already endorsed high levels of contraceptive acceptance, particularly given the high contraceptive use rates reported at baseline. Family planning research studies and programs are common in the study area, which may have also had an impact on the high contraceptive prevalence among the population we sampled from. Future research may consider limiting eligibility to non-contracepting men who do not desire pregnancy in the near future. Finally, future work with our scale items might work to better differentiate respondents at the highest levels, perhaps through use of different response categories.

Overall, the MNFPA scale is a promising contribution to the ongoing work to positively and meaningfully engage men in efforts to improve SRH and strengthen women’s reproductive autonomy. The scale may facilitate more valid evaluations of interventions that seek to transform male gender norms to embody more contraceptive acceptance and gender equity. Future researchers and program developers should continue to explore these norms as well as the multifaceted relationship between masculinity, contraceptive acceptance, and women’s contraceptive preferences and use.

## References

[CR1] Adams, R. J., Wu, M. L., Macaskill, G., Haldane, S. A., & Sun, X. X. (2016). *ACER ConQuest version 4.5.0: Generalized item response modeling software*. Camberwell, Australia: Australian Council for Educational Research and Berkeley: University of California (2016).

[CR2] Ajzen I (1991). The theory of planned behavior. Organizational Behavior and Human Decision Processes.

[CR3] American Educational Research Association (AERA), American Psychological Association (APA), and National Council for Measurement in Psychology (NCME) (2014). Standards for educational and psychological testing.

[CR4] Apanga PA, Adam MA (2015). Factors influencing the uptake of family planning services in the Talensi District Ghana. The Pan African Medical Journal.

[CR5] Bandura A (1989). Human agency in social cognitive theory. American Psychologist.

[CR6] Barker G, Ricardo C, Nascimento M, Olukoya A, Santos C (2010). Questioning gender norms with men to improve health outcomes: Evidence of impact. Global Public Health.

[CR7] de Boeck P, Wilson M (2004). Explanatory item response models: A generalized linear and nonlinear approach.

[CR8] Connell RW (1987). Gender and power.

[CR9] Costenbader E, Lenzi R, Hershow RB, Ashburn K, McCarraher DR (2017). Measurement of social norms affecting modern contraceptive use: A literature review. Studies in Family Planning.

[CR10] Courtenay WH (2000). Constructions of masculinity and their influence on men's well-being: A theory of gender and health. Social Science and Medicine.

[CR11] Cronbach LJ (1990). Essentials of psychologic testing.

[CR12] Dworkin S (2015). Men at risk: Masculinity, heterosexuality and HIV prevention.

[CR13] Dworkin SL, Colvin C, Hatcher A, Peacock D (2012). Men’s perceptions of women’s rights and changing gender relations in South Africa: Lessons for working with men and boys in HIV and antiviolence programs. Gender and Society.

[CR14] Dworkin SL, Dunbar MS, Krishnan S, Hatcher AM, Sawires S (2011). Uncovering tensions and capitalizing on synergies in HIV/AIDS and antiviolence programs. American Journal of Public Health.

[CR15] Dworkin SL, Hatcher AM, Colvin C, Peacock D (2013). Impact of a gender-transformative HIV and antiviolence program on gender ideologies and masculinities in two rural, South African communities. Men and Masculinities.

[CR16] Dworkin SL, Treves-Kagan S, Lippman SA (2013). Gender-transformative interventions to reduce HIV risks and violence with heterosexually-active men: A review of the global evidence. AIDS and Behavior.

[CR17] Eliason S, Baiden F, Quansah-Asare G, Graham-Hayfron Y, Bonsu D, Phillips J, Awusabo-Asare K (2013). Factors influencing the intention of women in rural Ghana to adopt postpartum family planning. Reproductive Health.

[CR18] Embretson SE, Reise SP (2000). Item response theory for psychologists.

[CR19] Fleminggg PJ, DiClemente RJ, Barrington C (2016). Masculinity and HIV: Dimensions of masculine norms that contribute to men’s HIV-related sexual behaviors. AIDS and Behavior.

[CR20] Flemingggg PJ, Agnew-Brune C (2015). Current trends in the study of gender norms and health behaviors. Current Opinion in Psychology.

[CR21] Flemingggg PJ, Barrington C, Maman S, Lerebours L, Donastorg Y, Brito MO (2019). Competition and humiliation: How masculine norms shape men's sexual and violent behaviors. Men and Masculinities.

[CR22] Fleminggggg PJ, Andes KL, DiClemente RJ (2013). 'But I'm not like that': Young men's navigation of normative masculinities in a marginalised urban community in Paraguay. Culture, Health and Sexuality.

[CR23] Geleta D (2018). Femininity, masculinity and family planning decision-making among married men and women in rural Ethiopia: A qualitative study. Journal of African Studies and Development.

[CR24] Ghanotakis E, Hoke T, Wilcher R, Field S, Mercer S, Bobrow EA, Mandera I (2017). Evaluation of a male engagement intervention to transform gender norms and improve family planning and HIV service uptake in Kabale, Uganda. Global Public Health.

[CR25] Grossman D, Onono M, Newmann SJ, Blat C, Bukusi EA, Shade SB, Cohen CR (2013). Integration of family planning services into HIV care and treatment in Kenya: A cluster-randomized trial. AIDS.

[CR26] Guttmacher Institute (2017). *Adding it up: The costs and benefits of investing in sexual and reproductive health 2017, fact sheet.* New York: Author. Retrieved from https://www.guttmacher.org/fact-sheet/adding-it-up-contraception-mnh-2017

[CR27] Hardee K, Croce-Galis M, Gay J (2017). Are men well served by family planning programs?. Reproductive Health.

[CR28] Harringtnnon E, Withers M, Dworkin S, Kwena Z, Onono M, Grossman D, Newmann S (2013). Covert contraceptive use and relationship power dynamics among couples in Nyanza Province, Kenya. Contraception.

[CR29] Harrington E, Dworkin S, Withers M, Onono M, Kwena Z, Newmann SJ (2016). Gendered power dynamics and women’s negotiation of family planning in a high HIV prevalence setting: A qualitative study of couples in western Kenya. Culture, Health and Sexuality.

[CR30] Harrington EK, Newmann SJ, Onono M, Schwartz KD, Bukusi EA, Cohen CR, Grossman D (2012). Fertility intentions and interest in integrated family planning to HIV services among women living with HIV in Nyanza Province. Kenya: A qualitative study. Infectious Diseases in Obstetrics and Gynecology.

[CR31] Hartmann M, Gilles K, Shattuck D, Kerner B, Guest G (2012). Changes in couples' communication as a result of a male-involvement family planning intervention. Journal of Health Communication.

[CR32] Hays RD, Morales LS, Reise SP (2000). Item response theory and health outcomes measurement in the 21st century. Medical Care.

[CR33] Igras S, Diakité M, Lundgren R (2017). Moving from theory to practice: A participatory social network mapping approach to address unmet need for family planning in Benin. Global Public Health.

[CR34] Imbuki K, Todd CS, Stibich MA, Shaffer DN, Sinei SK (2010). Factors influencing contraceptive choice and discontinuation among HIV-positive women in Kericho, Kenya. African Journal of Reproductive Health.

[CR35] James-Hawkins L, Salazar K, Hennink MM, Song Ha V, Yount KM (2019). Norms of masculinity and the cultural narrative of intimate partner violence among men in Vietnam. Journal of Interpersonal Violence.

[CR36] Jordal M, Wijewardena K, Öhman A, Essén B, Olsson P (2015). 'Disrespectful men, disrespectable women': Men's perceptions on heterosexual relationships and premarital sex in a Sri Lankan free trade zone - a qualitative interview study. BMC International Health and Human Rights.

[CR37] Kabagenyi A, Jennings L, Reid A, Nalwadda G, Ntozi J, Atuyambe L (2014). Barriers to male involvement in contraceptive uptake and reproductive health services: A qualitative study of men and women's perceptions in two rural districts in Uganda. Reproductive Health.

[CR38] Kaiser HF (1960). The application of electronic computers to factor analysis. Educational and Psychological Measurement.

[CR39] Kenya National AIDS Control Council (2016). Kenya HIV county profiles 2016.

[CR40] Kenya National Bureau of Statistics, and ICF International (2014). Kenya demographic and health survey 2014.

[CR43] Koffi TB, Weidert K, Ouro Bitasse E, Mensah MAE, Emina J, Mensah S, Prata N (2018). Engaging men in family planning: Perspectives from married men in Lomé, Togo. Global Health, Science and Practice.

[CR44] Lapinski MK, Rimal RN (2005). An explication of social norms. Communication Theory.

[CR45] Longford NT, Holland PW, Thayer DT, Holland PW, Wainer H (1993). Stability of the MH D-DIF statistics across populations. Differential item functioning.

[CR46] Mandal M, Muralidharan A, Pappa S (2017). A review of measures of women’s empowerment and related gender constructs in family planning and maternal health program evaluations in low- and middle-income countries. BMC Pregnancy and Childbirth.

[CR47] Masters GN (1982). Rasch model for partial credit scoring. Psychometrika.

[CR48] Maternowska MC, Withers M, Brindis C (2014). Gender, masculinity and migration: Mexican men and reproductive health in the Californian context. Culture, Health and Sexuality.

[CR50] Morrell R (2002). Men, movements, and gender transformation in South Africa. Journal of Men’s Studies.

[CR51] Mosha I, Ruben R, Kakoko D (2013). Family planning decisions, perceptions and gender dynamics among couples in Mwanza, Tanzania: A qualitative study. BMC Public Health.

[CR52] Newmann SJ, Grossman D, Blat C, Onono M, Steinfeld R, Bukusi EA, Cohen CR (2013). Does integrating family planning into HIV care and treatment impact intention to use contraception? patient perspectives from HIV-infected individuals in Nyanza Province, Kenya. International Journal of Gynecology and Obstetrics.

[CR53] Newmann SJ, Mishra K, Onono M, Bukusi EA, Cohen CR, Gage O, Grossman D (2013). Providers' perspectives on provision of family planning to HIV-positive individuals in HIV care in Nyanza Province Kenya. AIDS Research and Treatment.

[CR54] Newmann, S. J. (2010).* Fertility desires and family planning decision-making among HIV-affected couples in Nyanza Province, Kenya*. Oral presentation at Men, Masculinities, and Family Planning in Africa, UCLA.

[CR55] Nketiah-Amponsah E, Arthur E, Abuosi A (2012). Correlates of contraceptive use among Ghanaian women of reproductive age 15–49 years). African Journal of Reproductive Health..

[CR56] Ntshebe O (2011). Contraceptive decisions and HIV/AIDS concerns among married couples in Malawi. Journal of Biosocial Science.

[CR57] Odimegwu C, Pallikadavath S, Adedini S (2013). The cost of being a man: Social and health consequences of Igbo masculinity. Culture, Health and Sexuality.

[CR58] Ogunjuyigbe PO, Ojofeitimi EO, Liasu A (2009). Spousal communication, changes in partner attitude, and contraceptive use among the Yorubas of Southwest Nigeria. Indian Journal of Community Medicine.

[CR59] Paek, I. (2002)*. Investigations of differential item functioning: Comparisons among approaches, and extension to a multidimensional context*. Unpublished doctoral dissertation, University of California, Berkeley 2002.

[CR60] Palamuleni ME (2013). Socio-economic and demographic factors affecting contraceptive use in Malawi. African Journal of Reproductive Health.

[CR61] Patel R, Baum S, Grossman D, Steinfeld R, Onono M, Cohen C, Newmann S (2014). HIV-positive men's experiences with integrated family planning and HIV services in western Kenya: Integration fosters male involvement. AIDS Patient Care and STDs.

[CR62] Ruane-McAteer E, Amin A, Hanratty J, Lynn F, van Willenswaard KC, Reid E, Lohan M (2019). Interventions addressing men, masculinities and gender equality in sexual and reproductive health and rights: An evidence gap map and review of reviews. BMJ Global Health.

[CR63] Schuler SR, Rottach E, Mukiri P (2011). Gender norms and family planning decision-making in Tanzania: A qualitative study. Journal of Public Health in Africa.

[CR64] Schulermm SR, Nanda G, Ramírez LF, Chen M (2015). Interactive workshops to promote gender equity and family planning in rural communities of Guatemala: Results of a community randomized study. Journal of Biosocial Science.

[CR65] Shattuck D, Kerner B, Gilles K, Hartmann M, Ng'ombe T, Guest G (2011). Encouraging contraceptive uptake by motivating men to communicate about family planning: The Malawi Male Motivator Project. American Journal of Public Health.

[CR66] Shefer T, Crawford M, Strebel A, Simbayi LC, Dwadwa-Henda N, Cloete A, Kalichman SC (2008). Gender, power and resistance to change among two communities in the Western Cape, South Africa. Feminism and Psychology.

[CR67] Sideris T (2004). ‘You have to change and you don't know how!’: Contesting what it means to be a man in a rural area of South Africa. African Studies.

[CR68] Silberschmidt M (2011). Disempowerment of men in rural and urban East Africa: Implications for male identity and sexual behavior. World Development.

[CR69] Sileo KM, Fielding-Miller R, Dworkin SL, Fleming PJ (2018). What role do masculine norms play in men's HIV testing in sub-Saharan Africa?: A scoping review. AIDS and Behavior.

[CR70] Sileo KM, Fielding-Miller R, Dworkin SL, Fleming PJ (2019). A scoping review on the role of masculine norms in men's engagement in the HIV care continuum in sub-Saharan Africa. AIDS Care.

[CR71] Starrs AM, Ezeh AC, Barker G, Basu A, Bertrand JT, Blum R, Ashford LS (2018). Accelerate progress-sexual and reproductive health and rights for all: Report of the Guttmacher-Lancet Commission. Lancet.

[CR72] Steinfeld RL, Newmann SJ, Onono M, Cohen CR, Bukusi EA, Grossman D (2013). Overcoming barriers to family planning through integration: Perspectives of HIV-positive men in Nyanza Province Kenya. AIDS Research and Treatment.

[CR73] Stern E, Buikema R (2013). The relational dynamics of hegemonic masculinity among South African men and women in the context of HIV. Culture, Health and Sexuality.

[CR100] Tao AR, Onono M, Baum S, Grossman D, Steinfeld R, Cohen CR, Bukusi EA, Newmann SJ (2015). Providers’ perspectives on male involvement in family planning in the context of a cluster randomized controlled trial evaluating integrating family planning into HIV care in Nyanza Province, Kenya. AIDS Care.

[CR74] Tumlinson K, Speizer IS, Davis JT, Fotso JC, Kuria P, Archer LH (2013). Partner communication, discordant fertility goals, and contraceptive use in urban Kenya. African Journal of Reproductive Health.

[CR75] Wegs C, Creanga AA, Galavotti C, Wamalwa E (2016). Community dialogue to shift social norms and enable family planning: An evaluation of the Family Planning Results Initiative in Kenya. PLoS ONE.

[CR76] Wekesa E, Coast E (2014). Fertility desires among men and women living with HIV/AIDS in Nairobi slums: A mixed methods study. PLoS ONE.

[CR77] Wentzell EA, Inhorn MC (2014). Reconceiving masculinity and 'men as partners' for ICPD beyond 2014: Insights from a Mexican HPV study. Global Public Health.

[CR79] Willie TC, Khondkaryan E, Callands T, Kershaw T (2018). "Think like a man": How sexual cultural scripting and masculinity influence changes in men's use of intimate partner violence. American Journal of Community Psychology.

[CR80] Wilson M (2005). Constructing measures: An item response modeling approach.

[CR81] Wilson M, Allen DD, Li JC (2006). Improving measurement in health education and health behavior research using item response modeling: Comparison with the classical test theory approach. Health Education Research.

[CR82] Wingood GM, DiClemente RJ (2000). Application of the theory of gender and power to examine HIV-related exposures, risk factors, and effective interventions for women. Health Education and Behavior.

[CR83] Withers M, Dworkin S, Harrington E, Kwena Z, Onono M, Bukusi E, Newmann SJ (2013). Fertility intentions among HIV-infected, sero-concordant couples in Nyanza Province, Kenya. Culture, Health and Sexuality.

[CR84] Withers M, Dworkin S, Zakaras JM, Onono M, Oyier B, Cohen C, Newmann SJ (2013). ‘Women now wear trousers’: Men’s perceptions of family planning use in the
context of changing gender relations in western Kenya. Culture, Health and Sexuality.

[CR85] Withers M, Dworkin S, Onono M, Oyier B, Grossman D, Cohen C, Newmann S (2015). Men’s perspectives on their role in family planning in Nyanza Province, Kenya. Studies in Family Planning.

[CR86] Wright BD, Masters GN (1982). Rating scale analysis.

[CR87] Wyrod R (2008). Between women’s rights and men’s authority: Masculinity and shifting discourses of gender difference in urban Uganda. Gender and Society.

